# Reply to “*Mirage de tuberculose* in the 21^st^ century”

**DOI:** 10.5588/pha.24.0030

**Published:** 2024-09-01

**Authors:** B. Patterson, R. Wood

**Affiliations:** ^1^ Amsterdam Institute for Global Health and Development, University of Amsterdam, Amsterdam, The Netherlands;; ^2^ Aerobiology and TB Research Unit, Desmond Tutu Health Foundation, Cape Town, South Africa;; ^3^ Institute of Infectious Disease and Molecular Medicine, Faculty of Health Sciences, University of Cape Town, Cape Town, South Africa.

**Keywords:** mirage de tuberculose, subclinical tuberculosis, respiratory bioaerosol, aerosolised *Mycobacterium tuberculosis*

We read with interest the excellent case report and literature review by Kaelin and colleagues, which highlight the clinical scenario of transient *Mycobacterium tuberculosis* (*Mtb*) culture positivity in the respiratory tract.^1^

We believe the *mirage de tuberculose* phenomenon may be more common than generally realised. Our findings, published recently in the *Proceedings of National Academies of Science*,^2^ found the transient presence of *Mtb* in the respiratory tract detected using a fluorescent viability probe (DMN-trehalose)^3^ applied to collected respiratory bioaerosol samples. We sampled 102 presumptive TB patients from a high TB burden community in South Africa presenting with respiratory and/or systemic symptoms. Mtb was detected in >90% of cases in the aerosol sample. This high proportion was also present in those who had a negative sputum-Xpert Ultra (Cepheid, Sunnyvale, CA, USA) and who were not diagnosed with TB (*n* = 50). In most individuals in this group (80%), both symptoms and Mtb bioaerosol positivity resolved over time and in the absence of TB treatment. This indicates the transient nature of the host interaction – and thereby meets the *mirage* definition.

The aerosolised Mtb phenotype detected in our study was notable for poor culturability and a low bacillary count, which was likely below the detection limit for Xpert Ultra. This is consistent with the features in the reported case of slow time to culture positivity and a negative TB polymerase chain reaction. An important caveat is that exhaled bioaerosol is a distinct sample type that accesses the peripheral lung compartment and is, therefore, potentially a different TB subpopulation than sputum.

We agree with the authors' suggestion that *mirage de tuberculose* likely represents an immune-controlled stage of Mtb infection. In our paper, we speculate that the process of immune control may also be driving the symptoms that lead to clinical presentation. We also agree that Mtb self-clearance and the lack of TB disease progression make the benefit of treatment to the patient uncertain.

As a clinical entity, *mirage de tuberculose* lies on the TB disease spectrum but falls outside of typical definitions of subclinical TB due to the presence of symptoms, minimal TB disease and short-lived *Mtb* detection. It may, however, be of public health significance since, if infectious, even low-level and intermittent aerosol positivity in a vast pool of individuals may contribute significantly to onward transmission.^4^ Furthermore, the cyclical nature of TB disease, as theorised by Drain and colleagues,^5^ may need to be reconsidered in light of the host-pathogen paradigm. This theory, which was perhaps more evident during the pre-chemotherapy era when the natural progression of untreated TB was observed, suggests that our understanding of TB dynamics could benefit from revision.
